# Association between Beta2-Adrenergic Receptor Agonists and the Risk of Vascular Complications in Diabetic Patients: A Population-Based Cohort Study

**DOI:** 10.3390/jcm8081145

**Published:** 2019-07-31

**Authors:** Hee Jeong Lee, Haekyung Lee, Song Hee Oh, Suyeon Park, Kwang-Young Jung, Hyoungnae Kim, Soon Hyo Kwon, Jin Seok Jeon, Dong Cheol Han, Hyunjin Noh

**Affiliations:** 1 Division of Nephrology, Department of Internal Medicine, Soon Chun Hyang University, 59 Daesagwan-ro, Yongsan-gu, Seoul 04401, Korea; 2 Department of Biostatistics, Soon Chun Hyang University Seoul Hospital, 59 Daesagwan-ro, Yongsan-gu, Seoul 04401, Korea; 3 Hyonam Kidney Laboratory, Soon Chun Hyang University, 59 Daesagwan-ro, Yongsan-gu, Seoul 04401, Korea

**Keywords:** diabetes, vascular complications, beta2-adrenergic receptor, macrophages

## Abstract

Beta2-adrenergic receptor (β2AR) agonists can have protective effects targeting macrophage activation, but research on human subjects has not been done. This study was designed to assess the relationship between the use of β2AR agonists and diabetic vascular complications. Using data from the Korean National Health Insurance Service, adults first diagnosed with diabetes in 2004 (*n* = 249,222) were followed up until 31 December 2015. Propensity score matching was performed between case and control groups (*n* = 5179 in each), and multivariate analysis was conducted. The β2AR agonist group was divided into quartiles according to the duration of β2AR agonist use. During the follow-up, the incidence of vascular complications gradually decreased as the duration of β2AR agonist administration increased. Multivariate analysis revealed that the hazard ratio for all composite vascular complications was 0.80 (95% CI, 0.75–0.86, *p* < 0.001) in the longest quartile of β2AR agonist use as compared with the control group after adjusting for confounding variables. The association between the duration of β2AR agonist use and the risk of each vascular complication including cerebrovascular, peripheral vascular, peripheral neural, renal, and ophthalmic complications was consistent, and the risks were significantly lower in the longest users than the control group. Long-term use of β2AR agonists may exert a protective effect against diabetic vascular complications.

## 1. Introduction

Diabetes is associated with multiple vascular diseases that affect almost every arterial vascular bed [1]. Proatherogenic changes in diabetic patients include increases in vascular inflammation and derangements in the cellular components of the vasculature, most notably, endothelial cells and vascular smooth muscle cells [1]. Although the pathophysiology is not fully understood, macrophages are known to be significantly involved in this inflammatory process [2,3]. Previous studies have reported an association between macrophage differentiation and diabetic retinopathy [4], nephropathy [5], and coronary vascular diseases [2]. As a result, adults with diabetes have a two- to three-fold increased risk of a wide range of vascular diseases, including coronary heart disease and stroke [6]. Moreover, the odds ratios for diabetic patients with chronic kidney disease (CKD) vary by region between 1.3 and 4.6 [7].

Beta2-adrenergic receptor (β2AR) agonists are bronchodilators commonly used to treat bronchial asthma, chronic obstructive pulmonary disease (COPD), and other respiratory infections. β2AR agonists target and activate β2ARs to stimulate their downstream cascades, which mediate numerous airway functions by regulating bronchoconstriction and dilation pathways. β2AR agonists are very commonly used clinically and mainly lead to nonfatal complications such as headache, dizziness, and muscle cramps [8] without serious adverse effects.

In our own previous study, we identified β2AR agonists as the most potent anti-inflammatory drug in a multidrug screening analysis [9]. We confirmed their anti-inflammatory actions through multiple observations, including their inhibition of phorbol myristate acetate and lipopolysaccharide-induced tumor necrosis factor alpha (TNF-α) production in rat bone marrow macrophages, diabetes-induced TNF-α production in peripheral blood mononuclear cells from streptozotocin-induced diabetic rats, and macrophage infiltration in the kidneys and heart of Zucker diabetic fatty rats. Their mechanism of action involves inhibition of the nuclear factor κB (NF-κB) signal pathway by enhancing β-arrestin2 and its interaction with inhibitor of NF-κBα. From this study, we inferred that β2AR agonists might have preventive effects against vascular complications in diabetic patients. However, clinical studies demonstrating these anticipated effects of β2AR agonists have not yet been published. Our objective in this study was to determine whether β2AR agonists effectively inhibit macro- and microvascular complications in patients with diabetes.

## 2. Experimental Section

### 2.1. Study Design and Participants

This study cohort was comprised of Korean patients who were first diagnosed with diabetes in 2004 (*n* = 249,222). We defined this group as patients aged ≥20 years who received diagnostic codes for diabetes in 2004 along with more than two prescriptions for diabetes-related medications. To clarify the definition of new-onset diabetes, we excluded patients with diabetes codes within the previous two years (2002–2003). We also excluded patients who had diabetes codes but did not take antidiabetic medication or received such medications only once, because we could not be sure that these patients were truly diabetic. Patients with vascular complications due to diabetes at the time of enrollment were also excluded.

We divided the participants into two groups: the β2AR agonist group, including patients who received prescriptions for systemic β2AR agonists (oral administration) more than twice in 2004, and the control group including patients who did not take β2AR agonists during the study period (2004–2015). Systemic β2AR agonists included short-acting β2AR agonists (SABA), i.e., fenoterol, procaterol, salbutamol, terbutaline or long-acting β2AR agonists (LABA), i.e., bambuterol, formoterol, clenbuterol. To reduce confusion, we excluded patients who received prescriptions of systemic β2AR agonists only once in 2004 and patients who did not take systemic β2AR agonists in 2004 but received them later in the study period. In addition, patients who took β2AR agonists but died during the first two years of the study period (2004–2005) were excluded because those deaths were considered unrelated to the β2AR agonists. All subsequent deaths were registered in the study. In the end, we excluded 213,828 of the original (*n* = 249,222) participants, leaving a total of 35,245 (β2AR agonist group, *n* = 5179 and control group, *n* = 30,066).

**Figure 1:**
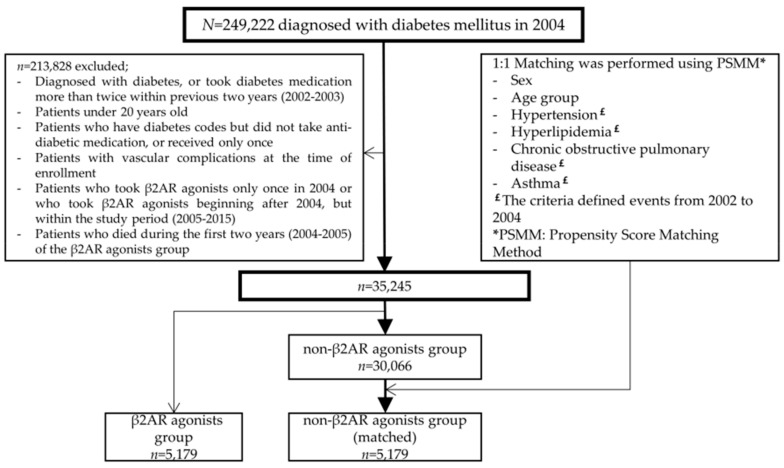
Flow chart of the study population. From the 249,222 participants initially enrolled, 213,828 patients were excluded. After 1:1 matching using propensity score matching, each group contained 5179 participants. Abbreviation: β2AR, beta2-adrenergic receptor.

We performed 1:1 matching using the propensity score matching method to ensure equivalence in sex, age, history of hypertension, hyperlipidemia, COPD, and asthma. History of hypertension or hyperlipidemia were defined as patients with the diagnostic codes for hypertension or hyperlipidemia, with corresponding medications prescribed more than twice per year during 2002–2004. A total of 5179 patients were included in each group and observed until 31 December 2015. No other clinical information about the patients was provided. After the 1:1 matching, the β2AR agonist group was classified into quartiles and analyzed according to the duration of β2AR agonist use. The flow chart of this study is shown in Figure 1.

### 2.2. Data Source and Ethics Statement

Data were extracted from the Korean National Health Insurance Service (KNHIS). All Koreans are registered in the KNHIS database, which has covered the entire population as a compulsory social insurance scheme since 1989 and is regulated by the Ministry of Health and Welfare [10]. Consequently, a large amount of health-related data has been accumulated in the KNHIS database, including data on demographic factors (age, sex, residential area, and socioeconomic variables such as type of health insurance) and medical factors (diagnosis codes, history of procedures or surgeries, and drug prescriptions). The KNHIS stores and manages the data and provides the data for research purposes [11]. More details about KNHIS have been provided elsewhere [10]. In the case of a disease covered by insurance in Korea, all clinicians must enter the diagnosis code and their prescriptions. Patient diagnoses are based on the International Statistical Classification of Diseases and Related Health Problems, 10th Revision (ICD-10-CM) diagnostic and procedure codes. We extracted the data as a custom sample, using the proposed conditions, i.e., patients diagnosed with diabetes in 2004.

This study protocol was approved by the institutional review board (IRB) of the Soon Chun Hyang University Seoul Hospital (IRB number 2017-05-001) and conducted in accordance with the Declaration of Helsinki. This was an anonymous observational study, so the need for informed consent was waived.

### 2.3. Outcome Measurements

The primary outcome was the occurrence of diabetic vascular complications. Complications were classified into two major categories of macro- and microvascular complications. Macrovascular complications included cardiovascular, cerebrovascular, and peripheral vascular complications; microvascular complications included peripheral neuropathy and renal and ophthalmic complications. Cardiovascular complications were defined as patients with diagnostic codes corresponding to coronary disease and its complications. Heart failure resulting from other myopathies was excluded. Cerebrovascular complications included occlusion and stenosis of cerebrovascular structures and diagnostic codes for stroke, excluding hemorrhagic stroke. Peripheral vascular complications included atherosclerosis, stenosis, and occlusion of peripheral arteries, along with symptomatic diagnostic codes such as claudication. Diabetic ulcer disease was also included. Renal complications included CKD in all stages, proteinuria including albuminuria, and end-stage renal disease. Peripheral neuropathy included diagnostic codes for various mono- and polyneuropathies that correspond to diabetic complications. Ophthalmic complications included various retinal and macular findings corresponding to diabetic complications, including neovascularization, aneurysm, edema, and hemorrhage.

Endpoints were assigned to each patient according to the diagnostic codes provided for complications. The disease-free survival time was calculated between the date of diabetes diagnosis and the date of diabetic complication diagnosis.

### 2.4. Statistical Analysis

To identify the effect of β2AR agonists, we used propensity score matching (Matchit in R package) to reduce the selection bias between groups. The unmatched variables (sex, age, hyperlipidemia, COPD, and asthma) were adjusted through multivariate analysis. A multivariate Cox proportional hazard regression analysis was performed by selecting factors with a significance level of less than 0.1 in the univariate analyses. Analysis was performed including the duration of β2AR agonist administration by dividing the β2AR agonist group into quartiles using the period (days) variable based on the number of β2AR agonist prescription days from enrollment in 2004 to the diagnosis of vascular complications. The Kaplan–Meier (KM) survival curve was plotted using SAS 9.3. All results were presented as a hazard ratio (HR) with a 95% confidence interval (CI). The level of significance was set at *p* < 0.05, and the tests were two-sided tests. All analyses were performed in SAS 9.3 and R v3.3.1.

## 3. Results

The baseline characteristics of the before and after matched groups are shown in Table 1. More female and elderly (aged ≥60 years) patients and patients with COPD or asthma were included in the β2AR agonist group even after 1:1 matching using the propensity score matching method. Meanwhile, the number of patients with a history of hyperlipidemia was lower in the β2AR agonist group. We analyzed the diagnostic codes at the time of β2AR agonist prescription and found that acute bronchiolitis was the most common code, followed by asthma and allergic rhinitis.

To determine the relationship between the occurrence of all vascular complications and the β2AR agonist administration along with its duration, we evaluated the incidence of all vascular complications within the β2AR agonist group according to the duration of β2AR agonist administration and then made comparisons between each of those groups and the control group. We classified the duration of β2AR agonist administration into quartiles. The follow-up period varied between complications because each endpoint was set differently for each complication. Therefore, quartile values differed for each complication. When analyzing the HR, we used multivariate Cox proportional hazard regression analysis to adjust for the relevant factors, including those not fully matched to increase accuracy.

**Figure 2:**
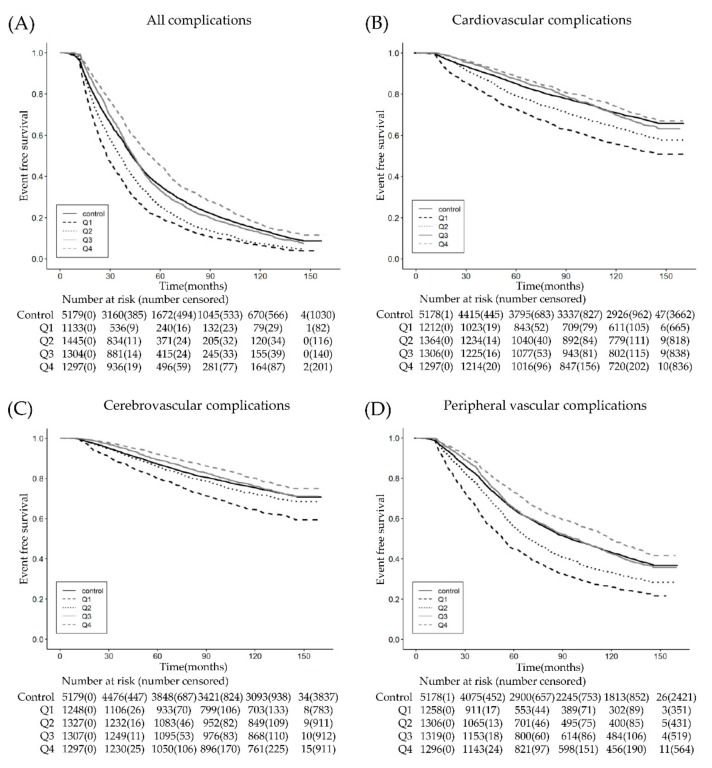

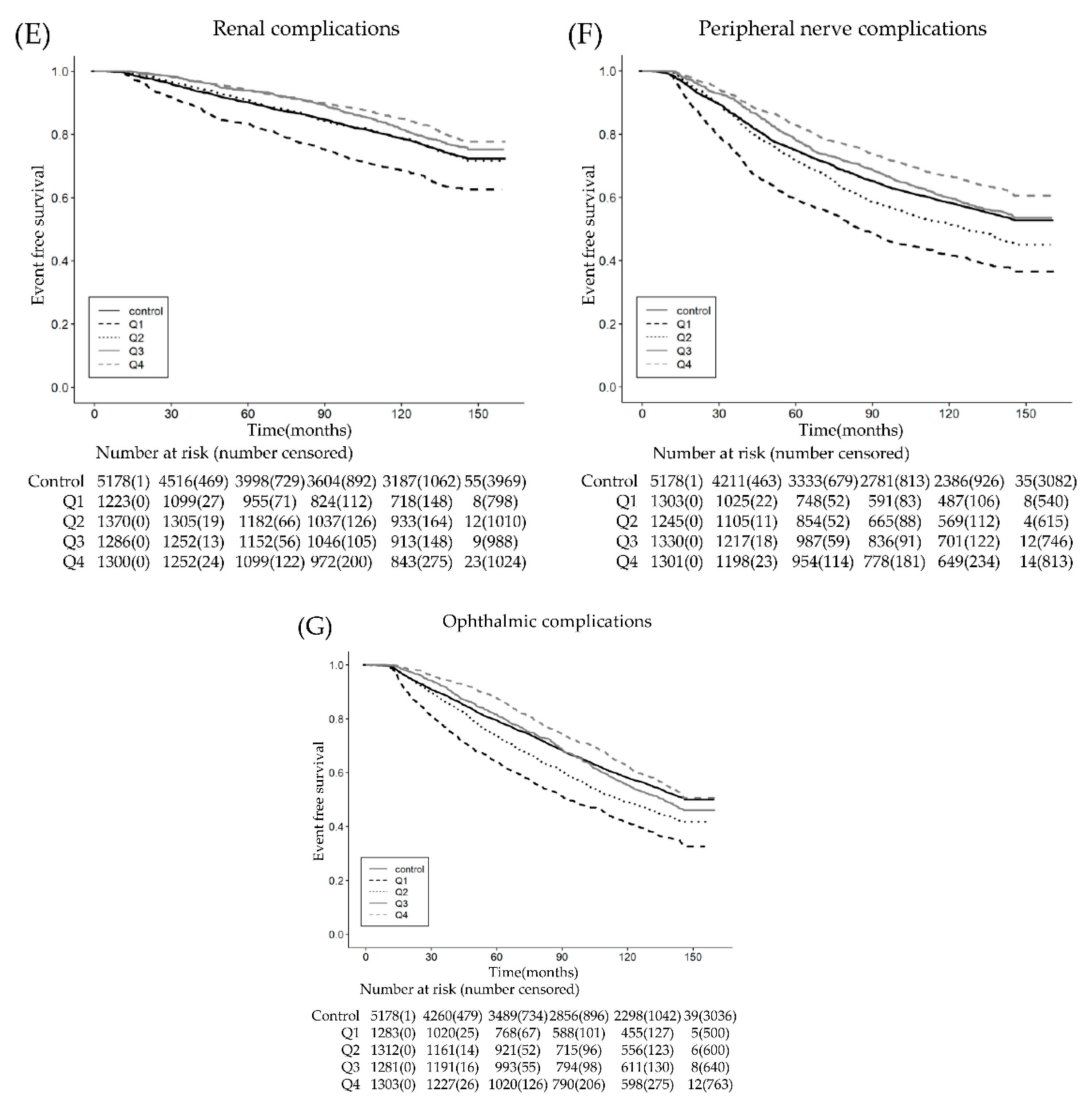
Kaplan–Meier survival curves comparing the outcomes for (**A**) all vascular complications, (**B**) cardiovascular complications, (**C**) cerebrovascular complications, (**D**) peripheral vascular complications, (**E**) renal complications, (**F**) peripheral nerve complications, and (**G**) ophthalmic complications according to the duration of β2AR agonist administration. The duration of the β2AR agonist administration was classified into quartiles. Abbreviation: β2AR, beta2-adrenergic receptor.

Upon Cox proportional hazard regression analysis by duration, the incidence of vascular complications significantly decreased with an increased duration of β2AR agonist use. The HRs for all vascular complications in quartiles 1 and 2 showed a higher risk of all vascular complications as compared with the non-β2AR agonist group (Table 2). However, in quartile 3, HR did not show any difference as compared with the non-β2AR agonist group. Furthermore, in quartile 4, the risk of all vascular complications was significantly reduced with an HR of 0.80 (95% CI, 0.75–0.86, *p* < 0.001). When analyzed separately for each vascular complication, cerebrovascular (HR 0.79; 95% CI, 0.69–0.89, *p* < 0.001), peripheral vascular (HR 0.82; 95% CI, 0.75–0.89, *p* < 0.001), peripheral nerve (HR 0.75; 95% CI, 0.67–0.83, *p* < 0.001), and ophthalmic complications (HR 0.88; 95% CI, 0.80–0.97, *p* = 0.008) all showed similar results, with all HRs in quartile 4 less than one. Of note, the risk of renal complications was significantly reduced as compared with the non-β2AR agonist group even in quartile 3 (HR 0.84; 95% CI, 0.74–0.96, *p* = 0.009). The risk of cardiovascular complications was qualitatively similar to that observed in the other complications except that the HR in quartile 4 did not achieve statistical significance (HR 0.90; 95% CI, 0.80–1.01, *p* = 0.068). The duration corresponding to quartile 4 was ≥42 days for all composite complications, ≥68 days for cerebrovascular complications, ≥55 days for peripheral vascular complications, and ≥62 days for peripheral nerve and ophthalmic complications. In terms of the renal complications, a protective effect was observed in the patients in quartile 3 who had been taking β2AR agonists for ≥28 days. The KM survival curves of these analyses are shown in Figure 2.

## 4. Discussion

In this study, we found the occurrence of vascular complications of diabetic patients significantly decreased with an increased duration of β2AR agonist administration, and eventually the risks were significantly lower than those in the non-β2AR agonist group for the longest users.

In our previous report, a drug screen analysis revealed that β2AR agonists have an anti-inflammatory action that targets macrophage activation in diabetic animals, especially in the kidneys and heart [9]. While a few studies have reported β2AR agonists as potential therapeutics for diabetic vasculopathy [12,13,14] in animal models, to the best of our knowledge, no human trials have been reported. We conducted this study to examine the association between β2AR agonists and the outcomes of diabetic macro- and microvascular complications, and to investigate whether β2AR agonists exert protective effects against these complications clinically, as observed in animal studies.

β2AR agonists are commonly used in acute respiratory infections, COPD, and asthma, a prescription pattern that was confirmed in this study by our examination of the common diagnostic codes among patients who received β2AR agonists. The present study included oral β2AR agonists, which are grouped into SABA and LABA. In general, COPD and asthma are associated with a higher prevalence of vascular diseases [15]. In particular, COPD patients are nearly five times more likely to have a cardiovascular disease than individuals without COPD [16]. It is known that chronic persistent inflammation in the respiratory tract and systemic circulation can stimulate inflammatory cells and modify various proteins, thereby activating atherosclerotic plaques and inducing a prothrombotic state [16]. Moreover, acute respiratory infections such as influenza or community-acquired pneumonia are also known to be associated with an increased risk of cardiovascular disease [17,18]. In a case-control study, recent respiratory infection has shown high associations with myocardial infarction (MI) and stroke; the odds ratio in the seven days following infection was 2.10 (1.38–3.21) for MI and 1.92 (1.24–2.97) for stroke [19]. In other words, most of the reasons for prescribing β2AR agonists are also common risk factors for cardiovascular diseases. In addition, it has been reported that COPD is associated with diabetic microvascular complications such as retinopathy [20], neuropathy [21], and CKD [22], although the associations between microvascular complications and acute respiratory infections remain unknown. Therefore, it is expected that the reasons for taking β2AR agonist are already the risk factors for various vascular complications. Our findings that the patients in the first or second quartile (short-term users) showed higher HRs as compared with the control group may be attributable to these findings. However, the important findings are that the risk of vascular complications gradually decreased with increasing duration of β2AR agonist administration and that the longest users in the fourth quartile showed a significantly reduced risk as compared to non-users. Given that the prolonged use of β2AR agonists is associated with sustained or severe disease activity due to COPD, asthma, or other respiratory infections, it is notable that the long-term use of β2AR agonists was associated with a significantly decreased incidence of all vascular complications as compared with the non-users. In fact, subjects in quartile 4 had a significantly higher prevalence of asthma and COPD and higher rates of elderly than the control group as shown in Supplementary Table S1. These findings suggest that the long-term use of β2AR agonists may exert a protective effect against diabetic vascular complications.

Additional analysis of each macro- and microvascular complication also confirmed the gradual decrease in the HRs as the duration increased. The time to achieve the beneficial effect of β2AR agonists on the composite, cerebrovascular, peripheral vascular, renal, peripheral nerve, and ophthalmic complications was 42, 68, 55, 28, 62, and 62 days, respectively. Interestingly, it is likely that the protective effect of β2AR agonists is most powerful against renal complications, since the patients in quartile 3, as well as in quartile 4, showed a significantly lower risk of such complications than the control group.

In general, it is well established that diabetic patients with macrovascular complications often have coincident microvascular complications [23,24,25]. However, independent associations between vascular complications and their differential regulation remain unclear. A recent study suggested that there may be shared protective factors for proliferative retinopathy and cardiovascular disease but not kidney disease in type 1 diabetes [26]. Another study has also shown that the presence of coronary artery calcification in long-standing type 1 diabetic patients was associated with neuropathy and retinopathy but not with renal hemodynamic function [27]. In the present study, however, we did not observe significant differences in the effects of long-term use of β2AR agonists according to the type of vascular complications other than a temporal relationship in renal complications.

To our knowledge, this is the first study supporting the effect of β2AR agonists on various vascular complications in diabetic patients. This was a population-based cohort study [28] with a long-term follow-up of more than 10 years from the diagnosis of diabetes.

However, our study also has some limitations. First, there was an absence of clinical information regarding smoking status, alcohol consumption, and documented history of hypertension, hyperlipidemia, COPD, or asthma, as we used only diagnostic codes to infer medical history information. This is a limitation of big data analysis in general. We attempted to address this problem by recruiting only those patients to whom a drug relevant to the diagnostic codes was prescribed at least twice. Second, we had no information as to the cause of death, which might have facilitated a more accurate assessment. Finally, the main limitation of our study was that we only analyzed patients taking oral medication and did not include inhaler users because the number of puffs prescribed varied according to the severity of symptoms, which made it difficult to confirm the exact duration of β2AR agonist use. This limitation may have caused the majority of β2AR agonist users to be excluded.

In conclusion, the incidence of vascular complications gradually decreased as the duration of β2AR agonist administration increased and, when taken for a sufficient period of time, the incidence was even lower than that in the control group. Our findings suggest that the long-term use of β2AR agonists may exert a protective effect against diabetic vascular complications. Further studies including inhaler users and well-designed human trials are warranted to elucidate the impact of β2AR agonists on vascular complications.

## Figures and Tables

**Table 1:** Baseline characteristics of participants before and after propensity score matching.

Variables		β2AR Agonist Group (*n* = 5179)		Non-β2AR Agonist Group				*p*-Value	
				Before Matching (*n* = 30,066)		After Matching (*n* = 5179)			
		*n*	%	*n*	%	*n*	%	Before	After
Sex	Male	2577	49.8	21,320	70.9	2724	52.6	<0.001	0.004
	Female	2602	50.2	8746	29.1	2455	47.4		
Age (years)	20~39	491	9.5	5057	16.8	596	11.5	<0.001	<0.001
	40~59	2287	44.2	17,630	58.6	2375	45.9		
	≥60	2401	46.4	7379	24.5	2208	42.6		
Hypertension	No	2870	55.4	20,371	67.8	2882	55.6	<0.001	0.812
	Yes	2309	44.6	9695	32.2	2297	44.4		
Hyperlipidemia	No	4381	84.6	26,261	87.3	4274	82.5	<0001	0.005
	Yes	798	15.4	3805	12.7	905	17.5		
COPD	No	3381	65.3	28,405	94.5	3518	67.9	<0.001	0.004
	Yes	1798	34.7	1661	5.5	1661	32.1		
Asthma	No	2772	53.5	28,946	96.3	4059	78.4	<0.001	<0.001
	Yes	2407	46.5	1120	3.7	1120	21.6		

Abbreviations: β2AR, beta2-adrenergic receptor; COPD, chronic obstructive pulmonary disease.

**Table 2:** Hazard ratios of vascular complications by duration of β2AR agonist administration in the β2AR agonist group.

			Univariate				Multivariate			
			HR	95% CI		*p*-Value	HR	95% CI		*p*-Value
				Lower	Upper			Lower	Upper	
All complications *	Period (days)	control	reference				reference			
		<8	1.52	1.42	1.63	<0.001	1.56	1.46	1.67	<0.001
		<17	1.29	1.21	1.37	<0.001	1.30	1.22	1.38	<0.001
		<42	1.06	1.00	1.13	0.069	1.01	0.95	1.08	0.740
		42≤	0.92	0.87	0.99	0.021	0.80	0.75	0.86	<0.001
Cardiovascular complications †	Period (days)	control	reference				reference			
		<11	1.65	1.50	1.82	<0.001	1.72	1.56	1.90	<0.001
		<25	1.34	1.21	1.48	<0.001	1.32	1.19	1.45	<0.001
		<67	1.11	1.00	1.24	0.046	1.04	0.93	1.15	0.5
		67≤	1.16	1.05	1.29	0.005	0.90	0.80	1.01	0.068
Cerebrovascular complications ‡	Period (days)	control	reference				reference			
		<12	1.46	1.31	1.62	<0.001	1.55	1.39	1.73	<0.001
		<26	1.12	1.00	1.25	0.045	1.11	0.99	1.24	0.066
		<68	1.04	0.93	1.17	0.483	0.96	0.85	1.07	0.440
		68≤	1.07	0.95	1.20	0.281	0.79	0.69	0.89	<0.001
Peripheral vascular complications §	Period (days)	control	reference				reference			
		<10	1.64	1.52	1.77	<0.001	1.68	1.56	1.82	<0.001
		<21	1.29	1.20	1.40	<0.001	1.29	1.20	1.40	<0.001
		<55	1.05	0.97	1.14	0.217	0.99	0.91	1.07	0.823
		55≤	0.96	0.89	1.05	0.381	0.82	0.75	0.89	<0.001
Renal complications ¥	Period (days)	control	reference				reference			
		<12	1.54	1.38	1.73	<0.001	1.54	1.37	1.72	<0.001
		<27	1.03	0.92	1.16	0.601	1.01	0.90	1.14	0.869
		<72	0.87	0.77	1.00	0.041	0.84	0.74	0.96	0.009
		72≤	0.81	0.70	0.92	0.002	0.75	0.65	0.86	<0.001
Peripheral nerve complications ¶	Period (days)	control	reference				reference			
		<11	1.60	1.47	1.73	<0.001	1.64	1.51	1.79	<0.001
		<23	1.20	1.10	1.31	<0.001	1.21	1.11	1.33	<0.001
		<62	0.94	0.86	1.04	0.222	0.93	0.85	1.02	0.126
		62≤	0.81	0.74	0.90	<0.001	0.75	0.67	0.83	<0.001
Ophthalmic complications #	Period (days)	control	reference				reference			
		<11	1.68	1.55	1.83	<0.001	1.70	1.57	1.85	<0.001
		<24	1.29	1.19	1.41	<0.001	1.29	1.18	1.40	<0.001
		<62	1.09	1.00	1.19	0.061	1.07	0.98	1.17	0.135
		62≤	0.90	0.82	0.99	0.027	0.88	0.80	0.97	0.008

Abbreviations: β2AR, beta2-adrenergic receptor; HR, hazard ratio; CI, confidence interval; COPD, chronic obstructive pulmonary disease. * *p*-value adjusted by β2AR agonist, sex, age, hypertension, hyperlipidemia, COPD, and asthma. † *p*-value adjusted by β2AR agonist, age, hypertension, hyperlipidemia, COPD, and asthma. ‡ *p*-value adjusted by β2AR agonist, sex, age, hypertension, hyperlipidemia, COPD, and asthma. § *p*-value adjusted by β2AR agonist, sex, age, hypertension, hyperlipidemia, COPD, and asthma. ¥ *p*-value adjusted by β2AR agonist, hyperlipidemia, and asthma. ¶ *p*-value adjusted by β2AR agonist, sex, age, hyperlipidemia, and COPD. # *p*-value adjusted by β2AR agonist, sex, age, and hyperlipidemia.

## Supplementary Materials

The following are available online at https://www.mdpi.com/2077-0383/8/8/1145/s1, Table S1: Baseline characteristics of β2AR agonist group by duration.
