# Supplementary material for: Association between Beta2-Adrenergic Receptor Agonists and the Risk of Vascular Complications in Diabetic Patients: A Population-Based Cohort Study
Source: J Clin Med. 2019 Jul 31;8(8):1145. doi: 10.3390/jcm8081145 (PMC6722988; doi:10.3390/jcm8081145)
Supplement: Supplementary file 1 [file jcm-08-01145-s001.pdf]

Table S1. Baseline characteristics of β2AR agonists group by duration

		Control group (*n* =5179)		Quartile 1 (*n* =1133)		Quartile 2 (*n* =1445)		Quartile 3 (*n* =1304)		Quartile 4 (*n* =1297)		*P* -value
		n	%	*n*	%	*n*	%	*n*	%	*n*	%	
**Sex**	Male	2724	52.6	561	49.5	705	48.8	604	46.3	707	54.5	
	Female	2455	47.4	572	50.5	740	51.2	700	53.7	590	45.5	<0.001
**Age**	20~39	596	11.5	141	12.4	162	11.2	122	9.4	66	5.1	
	40~59	2375	45.9	565	49.9	697	48.2	576	44.2	449	34.6	<0.001
	>60	2208	42.6	427	37.7	586	40.6	606	46.5	782	60.3	
**Hypertension**	No	2882	55.6	646	57.0	816	56.5	698	53.5	710	54.7	
	Yes	2297	44.4	487	43.0	629	43.5	606	46.5	587	45.3	0.405
**Hyperlipidemia**	No	4274	82.5	951	83.9	1211	83.8	1086	83.3	1133	87.4	
	Yes	905	17.5	182	16.1	234	16.2	218	16.7	164	12.6	0.001
**COPD**	No	3518	67.9	864	76.3	1092	75.6	877	67.3	548	42.3	
	Yes	1661	32.1	269	23.7	353	24.4	427	32.7	749	57.7	<0.001
**Asthma**	No	4059	78.4	771	68.0	916	63.4	694	53.2	391	30.1	
	Yes	1120	21.6	362	32.0	529	36.6	610	46.8	906	69.9	<0.001

Abbreviations: β2AR, beta2-adrenergic receptor; COPD, chronic obstructive pulmonary disease.
